# The Role of Functional Risk and Fear of Falling in Older Adults’ Everyday Walking Activity

**DOI:** 10.1093/geroni/igaa057.968

**Published:** 2020-12-16

**Authors:** Carl-Philipp Jansen, Jochen Klenk, Corinna Nerz, Sarah Labudek, Franziska Kramer, Clemens Becker, Michael Schwenk

**Affiliations:** 1 Heidelberg University, Karlsruhe, Germany; 2 Robert-Bosch Hospital Stuttgart, Stuttgart, Germany; 3 Heidelberg University, Heidelberg, Germany

## Abstract

Some persons have low functional risk (FR) but also high levels of fear of falling (FOF), in some it may be the exact opposite; in others, FOF matches actual functional risk. In order to characterise older persons in this respect, Delbaere et al. (2010) defined four groups: ‘vigorous’ (low FR/FOF), ‘anxious’ (low FR/high FOF), ‘stoic’ (high FR/low FOF), and ‘aware’ (high FR/FOF). We examined how the proposed group model translates into actual walking behaviour and explored whether group differences in walking occur due to FR level rather than the amount of FOF. Group allocation of N=294 participants was determined based on previously published cut-offs for FR (high vs. low Timed Up-and-Go) and FOF (high vs. low Short Falls-Efficacy Scale International). Walking activity was operationalised as mean number of steps per day over one week, assessed using ‘activPAL4™ micro’ accelerometers. Number of steps in the four groups were 6,335 (‘vigorous’), 5,782 (‘anxious’), 4,851 (‘stoic’), and 4,627 (‘aware’). Linear regression results showed that in the two low FR groups, those with high FOF did not differ significantly from the reference group with low FOF (anxious - vigorous: B=-645.3 steps, p=.157); however, the two groups with high FR showed a significantly different number of steps than the ‘vigorous’ group, irrespective of their FOF (aware-vigorous: B=-1536.1 steps, p=.002; stoic-vigorous: B=-1314.8 steps, p=.005). This means that FR outperformed FOF in their association with walking behaviour, i.e., participants can be better separated in their daily walking behaviour by FR than by FOF.

